# Metabolically Healthy Obesity Is Characterized by a Distinct Proteome Signature

**DOI:** 10.3390/ijms26052262

**Published:** 2025-03-04

**Authors:** Fayaz Ahmad Mir, Houari B. Abdesselem, Farhan Cyprian, Ahmad Iskandarani, Asmma Doudin, Mutasem AbdelRahim Shraim, Bader M. Alkhalaf, Meis Alkasem, Ibrahem Abdalhakam, Ilham Bensmail, Hamza A. Al Halabi, Shahrad Taheri, Abdul-Badi Abou-Samra

**Affiliations:** 1 Qatar Metabolic Institute, Academic Health System, Hamad Medical Corporation, Doha P.O. Box 3010, Qatar; aiskandarani@hamad.qa (A.I.); balkhalaf@hamad.qa (B.M.A.); iabdalhakam@hamad.qa (I.A.); hamzauniversity2@gmail.com (H.A.A.H.); asamra@hamad.qa (A.-B.A.-S.); 2 College of Medicine, QU Health, Qatar University, Doha P.O. Box 2713, Qatar; fcyprian@qu.edu.qa; 3 Proteomics Core Facility, Qatar Biomedical Research Institute (QBRI), Hamad Bin Khalifa University (HBKU), Qatar Foundation, Doha P.O. Box 34110, Qatar; 4 Laboratory of Immunoregulation, Research Department, Sidra Medicine, Doha P.O. Box 26999, Qatar; asmaa.doudin@hotmail.com; 5 National Obesity Treatment Center, Hamad Medical Corporation, Doha P.O. Box 3010, Qatar; 6 Weil Cornell Medicine—Qatar, Doha P.O. Box 24144, Qatar

**Keywords:** obesity, proteomics, Olink, comorbidities

## Abstract

Obesity is commonly associated with metabolic diseases including type 2 diabetes, hypertension, and dyslipidemia. Moreover, individuals with obesity are at increased risk of cardiovascular disease. However, a subgroup of individuals within the obese population presents without concurrent metabolic disorders. Even though this group has a stable metabolic status and does not exhibit overt metabolic disease, this status may be transient; these individuals may have subclinical metabolic derangements. To investigate the latter hypothesis, an analysis of the proteome signature was conducted. Plasma samples from 27 subjects with obesity but without an associated metabolic disorder (obesity only (OBO)) and 15 lean healthy control (LHC) subjects were examined. Fasting samples were subjected to Olink proteomics analysis targeting 184 proteins enriched in cardiometabolic and inflammation pathways. Our results distinctly delineated two groups with distinct plasma protein expression profiles. Specifically, a total of 24 proteins were differentially expressed in individuals with obesity compared to LHC. Among these, 13 proteins were downregulated, whereas 11 proteins were upregulated. The pathways that were upregulated in the OBO group were related to chemoattractant activity, growth factor activity, G protein-coupled receptor binding, chemokine activity, and cytokine activity, whereas the pathways that were downregulated include regulation of T cell differentiation, leukocyte differentiation, reproductive system development, inflammatory response, neutrophil, lymphocyte, monocyte and leukocyte chemotaxis, and neutrophil migration. The study identifies several pathways that are altered in individuals with obesity compared to healthy control subjects. These findings provide valuable insights into the underlying mechanisms, potentially paving the way for the identification of therapeutic targets aimed at improving metabolic health in individuals with obesity.

## 1. Introduction

The global prevalence of obesity is expected to rise from 14% to 24% during the period 2020–2035, affecting nearly 2 billion adults, children, and adolescents by 2035 [1]. Obesity is a chronic disease characterized by excess subcutaneous and visceral fat deposition. Obesity is a major risk factor for several disorders, such as cardiovascular, metabolic, neurodegenerative, musculoskeletal, hepatobiliary, reproductive, certain types of cancer, and endothelial dysfunction [2,3,4,5,6,7], and it reduces the quality of life [8]. Obesity dampens the strength of the immune system, thereby rendering people with obesity vulnerable to infectious diseases; several independent studies have provided powerful evidence that obesity is a risk factor for severe COVID-19 outcomes [9].

Obesity is a heterogeneous and complex condition. Individuals with obesity but without metabolic disorders have been described as having “healthy obesity” or “obesity only” (OBO) [10]. OBO may be a transitional condition that may harbor subclinical metabolic derangements. The advancement of biochemical analyses over the past 20 years has facilitated the endocrine and biochemical profiling of obesity [11,12,13,14]. This has brought about a subdivision in the concept of obesity by metabolic/biochemical dysfunction. Even though the OBO group has apparently stable metabolic status and possesses a low risk for certain metabolic diseases compared to their unhealthy counterparts, they are at increased risk for type 2 diabetes compared to LHC [15]. Multiple studies reported that the OBO phenotype has a normal lipid profile and normal or slightly impaired insulin sensitivity, despite a similar amount of body fat, and that OBO status is maintained for a long time [16,17]. The “OBO hypothesis” has been criticized as it is a temporary condition; sooner or later risk factors will become visible, and metabolic complications may develop in these patients [18]. A better insight into the biological pathways that are affected by excess body fat will allow early prognostication and the development of new strategies to protect against metabolic dysfunctions, preferably before symptoms are evident. Although previous studies have focused on characterizing metabolically healthy obesity (MHO) and its association with specific biomarkers, the proteomic mechanisms underlying this phenomenon remain inadequately explored, especially in populations with obesity but without overt metabolic abnormalities.

The technologies of high-throughput omics are evolving to comprehensively identify and quantify biomolecules in blood, body fluids, and tissues, providing a new avenue for dissecting pathophysiological mechanisms and discovering biomarkers in diseases [19]. Proteomics is a system biology approach for studying a group of proteins produced in cells, tissues, and body fluids. The impaired expression of proteins in a disease reflects abnormalities in the individual. Thus, measurable protein biomarkers are used for disease diagnosis or to indicate disease severity. Blood is the most widely utilized diagnostic sample due to its low invasiveness.

We hypothesized that despite the OBO group having an apparently stable metabolic status, this eventually evolves towards OBM, carrying an increased risk for cardiovascular disease and mortality over time. To investigate the changes occurring at the molecular level without any change in the phenotype of these OBO individuals, we investigated plasma proteomes using an Olink assay. The plasma proteome plays a crucial role in various biological processes including signal transmission, transport, growth, repair, and defense against infection [20]. Olink proteomics technology employs a proximity extension assay (PEA) combined with a bioinformatic analysis to measure the changes of inflammatory and cardiometabolic-related proteins in plasma samples in individuals with OBO and LHC. The rationale of this study was to identify the underlying metabolic pathways that are dysregulated in OBO subjects vs. LHC. Our study adds to the growing body of literature by identifying distinct proteomic profiles in individuals with obesity but without metabolic dysfunction, differentiating them from healthy controls. In contrast to previous studies that primarily focused on inflammatory and lipid-related biomarkers, our findings suggest that a broader range of proteomic pathways, including immune modulation and growth factor signaling, may contribute to the metabolic stability observed in this subgroup of individuals.

## 2. Results

### 2.1. Study Cohort

**Figure 1:**
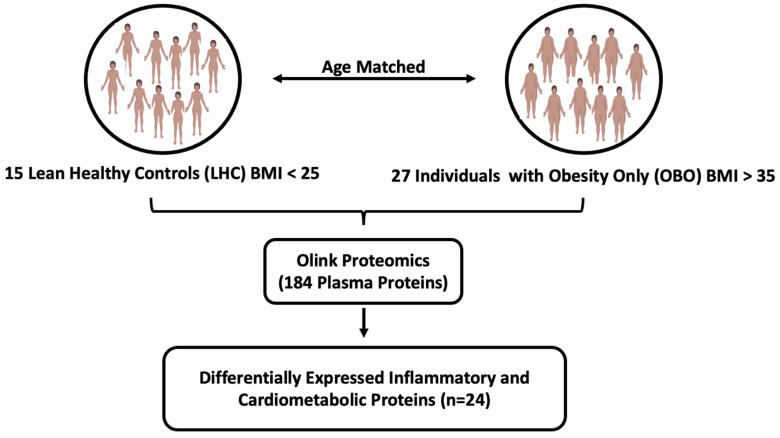
The experimental study designs. Abbreviations: BMI (body mass index), OBO (obesity only (no metabolic disease)), LHC (lean healthy controls).

Participants were classified into two groups: OBO and LHC. The OBO phenotype was defined as BMI ≥ 35 kg/m^2^ without clinical metabolic syndrome, whereas the LHC group participants had BMI < 27 kg/m^2^ and were not taking any medication for a chronic health condition. Samples from 27 OBO and 15 LHC subjects were selected for proteomic analyses (Figure 1). Among the 27 OBO subjects, 2 individuals had isolated hypertension, 2 had mildly elevated triglycerides, and 6 had borderline low HDL cholesterol (1.03 to 1.55 mmol/L) (Table 1).

### 2.2. Proteomic Analysis Revealed a Differential Plasma Protein Profile in Obesity

**Figure 2:**
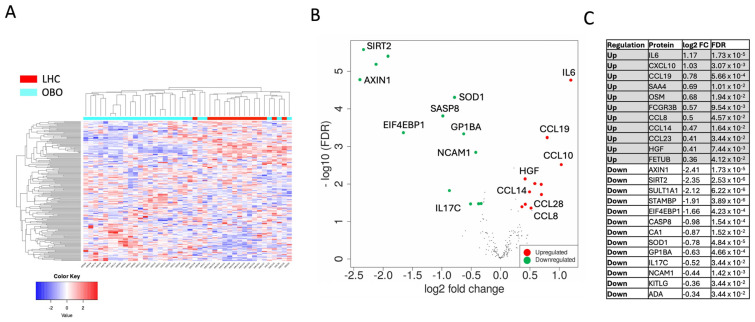
Differential protein expression analysis between OBO and LHC. Differentially expressed proteins (DEPs) were identified from combined Olink inflammation and cardiometabolic panels (184 unique proteins) defined as DEPs with more than a 1.25-fold change with FDR < 0.05. (**A**) Hierarchical clustering based on all 184 proteins assayed using the two Olink panels showed a separation between obese patients compared to controls, with a cutoff Z score of 4. (**B**) A volcano plot summarizing DEPs based on log2 fold changes across the two groups. The red and green circles show significantly differential expressed proteins ≥ 0.32 log2 fold change (upregulated) or ≥−0.32 log2 fold change (downregulated) and FDR < 0.05. Differential expression analysis accounted for obesity as the main effect, while correcting for age, sex, and race. (**C**) OBO signature protein list.

The proteomic analysis demonstrated a distinct plasma protein expression profile in OBO compared to LHC. The unsupervised hierarchical clustering revealed that the two panels differentiated between the OBO and LHC individuals, as demonstrated by the resulting heatmap (Figure 2A). A total of 24 proteins were differentially expressed in OBO subjects compared to LHC subjects (Figure 2B,C). Thirteen proteins were found to be downregulated, whereas 11 proteins were upregulated at a minimum fold change of 1.25 and a false discovery rate (FDR) cutoff of 0.05 (Supplementary Data S2).

As shown in Figure 2C, the 11 significantly upregulated proteins in OBO compared to LHC were Intelukin-6 (IL-6), C-X-C motif Chemokine ligand 10 (CXCL10), Chemokine Ligand 19 (CCL19), Serum Amyloid A4 (SAA4), Oncostatin M (OSM), Receptor for the Fc Region of Immunoglobulins gamma (FCGR3B), Chemokine (C-C motif) Ligand 8 (CCL8), Chemokine (C-C motif) Ligand 14 (CCL14), Chemokine (C-C motif) Ligand 23 (CCL23), Hepatocyte Growth Factor (HGF), and Fetuin B (FETUB). Thirteen proteins were significantly downregulated in OBO compared to LHC subjects: axis inhibition protein 1 (AXIN1), Sirtuin 2 (SIRT2), Sulfotransferase 1A1 (SULT1A1), STAM-binding protein (STAMBP), Eukaryotic translation initiation factor 4E-binding protein 1 (EIF4EBP1), Caspase-8 (CASP8), Carbonic anhydrase 1 (CA1), Superoxide dismutase 1 (SOD1), glycoprotein Ib platelet subunit alpha (GP1BA), Interleukin 17C (IL-17C), neural cell adhesion molecule 1 (NCAM1), Ligand for the receptor-type protein-tyrosine kinase KIT (KITLG), and alkyl guanine transferase I (ADA). To identify the interactions between DEPs and to gain insights into the protein network that is affected in OBO, a protein–protein interaction STRING analysis was performed on the DEPs in OBO cases (Figure 2A). Visualizing the interactions between DEPs revealed inflammatory protein networks orchestrated by IL-6, CXCL10, CCL13, and CCL2. Other protein–protein interaction networks are also displayed, such as Axin1, HGF, CASP8, NCAM1, and FCGR3B.

### 2.3. Functional Enrichment Analysis of DEPs Revealed Immunological Pathways in Obesity

**Figure 3:**
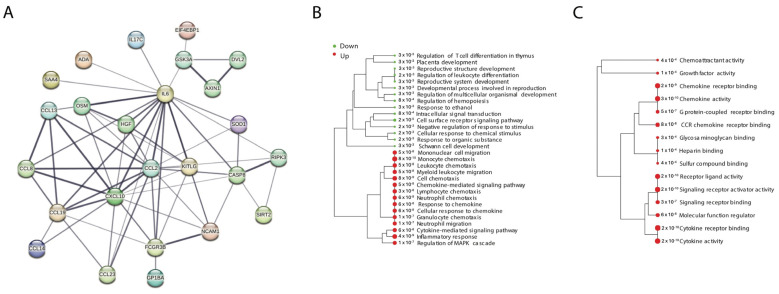
Protein–protein interactions and pathway analysis of DEPs in OBO versus LHC. (**A**) The 24 DEPs were run in the STRING network to assess their protein–protein interactions. (**B**,**C**) Pathway analysis was performed using Gene Ontology (GO): (**A**) shows GO biological processes and (**B**) GO molecular functions. The size of the dots corresponds to adjusted *p*-values for statistically significant upregulation or downregulation represented by the red and green circles, respectively.

A GO pathway analysis was performed to identify the functional contribution of DEPs in biological processes and molecular components. DEPs were found to be enriched in biological processes related to immune and reproductive systems. Analysis revealed that 15 pathways were upregulated, and 15 pathways were downregulated in OBO, such as regulation of T cell differentiation, leukocyte differentiation, reproductive system development, inflammatory response, neutrophil, lymphocyte, monocyte and leukocyte chemotaxis, and neutrophil migration (Figure 3B). Furthermore, 15 molecular pathways were upregulated in OBO cases (Figure 3C), including chemoattractant activity, growth factor activity, G protein-coupled receptor binding, chemokine activity, and cytokine activity (Supplementary Data S3).

## 3. Discussion

The associated metabolic changes make obesity a significant risk factor for various diseases, impacting both quality of life and mortality rates. This study sheds light on the complex nature of obesity, focusing on a subgroup termed “obesity only” (OBO), characterized by obesity without clinically apparent metabolic disorders. The OBO phenotype has been debated as a transitional state with comparatively lower health risks. Our investigation utilized the high-throughput Olink proteomics platform to examine the plasma proteome in individuals with OBO and compare it with that in LHC. Our results show a general increase in pro-inflammatory cytokines and chemokines (IL-6, CCL19, CXCL10, CCL14, CCL28, and CCL8) in the OBO cohort apart from IL17c, which is downregulated. Remarkably, individuals with OBO exhibit downregulation of key proteins associated with clotting and adhesion, namely GP1BA and NCAM, along with a reduction in the expression of specific cellular function regulators (SIRT2, STAMBP, SASP8, ELF4EBP1, AXIN1, and SOD1). In contrast to these downregulated proteins, the hepatic growth factor (HGF) is notably elevated in the OBO cohort (Figure 2).

The novel findings in our study lie in the identification of several previously unexplored proteins and pathways associated with the OBO phenotype. Specifically, we identified the downregulation of proteins involved in cellular protection and clotting, such as GP1BA and NCAM1, which may confer a protective effect by reducing microvascular clot formation in the OBO group [21]. Conversely, the upregulation of proteins such as HGF and CCL19, along with increased inflammatory cytokines like IL-6, CXCL10, and CCL8, suggests an inflammatory environment that could predispose OBO individuals to the development of a metabolic syndrome [22].

The controversial role of IL-6 in adipose tissue on obesity-induced dysregulation of glucose metabolism was reviewed recently by Wueest and Konrad [23]. IL6 is produced by adipose tissue and is associated with chronic low-grade inflammation. Elevated levels of IL-6 have been linked to insulin resistance, glucose metabolism, and the development of metabolic disorders. The elevated IL-6 observed in the OBO cohort correlates well with the reported clinical findings of elevated glucose, HbA1C, and insulin levels (Table 1 and Supplementary Figure S1A). Interestingly, elevated IL-6 corresponds to the increase in CRP reported in the OBO cohort. Indeed, trials involving IL-6 inhibition show a marked reduction in CRP and serum amyloid A [24,25,26]. These findings are consistent with the known inflammatory role of IL-6 in the setting of chronic low-grade inflammation observed in obese diabetic patients [27,28,29].

While transient elevation during fasting or exercise facilitates lipid mobilization, chronic elevation, predominantly originating from immune cells and adipocytes within white adipose tissue (WAT), can elicit pathological consequences. Prolonged high IL-6 levels exert endocrine influences, impacting gastric emptying, GLP-1 release, and hepatic insulin sensitivity, thereby perturbing glucose homeostasis. The modified secretory profile in WAT, mediated by IL-6 through autocrine or paracrine actions, further contributes to metabolic dysregulation. IL-6 exhibits context-dependent effects, demonstrating anti-inflammatory actions in macrophages and pro-inflammatory responses in T cells. Additionally, IL-6 stimulates the release of leptin and free fatty acids from adipocytes in a depot-specific manner. Indeed, several studies have indicated the role of chronic low-grade inflammation in neurodegenerative disorders such as Alzheimer’s disease (AD) and Parkinson’s disease (PD), in which the role of leptin has been highlighted [2,3,30,31].

Moreover, a recent study has documented elevated CCL19 levels in correlation with CRP among individuals with obesity, indicating a potential association with systemic inflammation [32]. The study further reveals cooperative relationships between CCL19 and factors such as adiponectin, plasma triglycerides, FBG, and HbA1c, shedding light on their collective impact on insulin resistance in obesity [32]. Similarly, elevated levels of CXCL10 and CXCL11 in obese individuals have been reported to impede neovascularization during adipose tissue (AT) expansion. Insufficient neovascularization during adipose tissue expansion is hypothesized to induce a hypoxic state, concomitant with heightened inflammation and metabolic dysfunction [33]. Notably, chemokines play a crucial role in obesity-induced insulin resistance, type 2 diabetes, and cardiovascular disease. Insulin resistance is further aggravated in the OBO cohort since SIRT2 suppression no longer inhibits adipogenesis (Supplementary Figure S1A) [34]. Moreover, chemokine networks are implicated in both local and systemic inflammations associated with adipose tissue-macrophage interaction [35]. Other chemokines that were found upregulated in the OBO group were CCL8 and CCL23, which promote the chemotaxis of leukocytes, while CCL14 specifically activates monocytes [36].

Additionally, serum amyloid A (SAA) subtypes 1–3, classic acute phase reactants elevated in acute inflammation, have been shown to exhibit associations with chronic metabolic diseases, including obesity, diabetes, and cardiovascular disease, underscoring the role of these inflammatory markers in the OBO group [37,38,39,40,41,42]. Chronic low-grade inflammation is a key driver of metabolic dysfunction in obesity. Furthermore, the receptor for the Fc region of immunoglobulin gamma (FCGR3B) was found to be upregulated in the OBO group. Fc gamma receptors (FcγRs) are pivotal for recognizing IgG-coated targets and facilitating antigen internalization. Altered FcγR biology in obesity could affect antigen processing, potentially perpetuating chronic inflammation and impacting immune responses.

Additionally, research has revealed elevated circulating levels of HGF in conditions such as obesity, metabolic syndrome, and diabetes mellitus. Notably, obese individuals exhibited more than a threefold increase in circulating HGF levels compared to their lean counterparts [43]. Another hepatokine we found elevated in the OBO group was Fetuin B, which is modulated through leptin–STAT3 signaling and is known to exhibit an association with leptin in the context of obesity [44].

Conversely, Oncostatin M (OSM), an IL-6 family cytokine that influences cell growth, neuronal development, and inflammatory responses, was downregulated in the OBO group. In mouse models, OSM receptor deficiency leads to late-onset obesity, adipose tissue inflammation, and insulin resistance [45]. A high-fat diet exacerbates metabolic disorders in these mice, while OSM treatment improves metabolic syndrome, altering adipose tissue macrophages towards an anti-inflammatory M2 phenotype and regulating hepatic lipid metabolism enzymes. Although OSM has been proposed as a novel therapeutic target for metabolic syndrome, further research is warranted to provide insights into its role in inflammation, insulin resistance, and lipid metabolism in the context of obesity-related metabolic syndrome.

Additionally, Ren et al. [46] demonstrated that Sirtuin 2 protects against liver steatosis and metabolic disorders through the deacetylation of hepatocyte nuclear factor 4α (HNF4α). Furthermore, genomic analysis revealed SIRT2 downregulation in advanced NAFLD patients and HFD-induced NAFLD mice. Obese mice and palmitate-treated cells exhibited decreased SIRT2 levels. Restoring hepatic SIRT2 mitigated insulin resistance, hepatic steatosis, and inflammation, while liver-specific ablation exacerbated dysfunctions. Targeting the SIRT2–HNF4α axis holds promise for treating fatty liver diseases and metabolic disorders in obese individuals [46].

Furthermore, studies have demonstrated the elevation of pro-inflammatory cytokines in diet-induced obesity leads to decreased levels of drug metabolism enzymes, including sulfotransferases. Whereas rodent models have indicated that lipopolysaccharide-induced inflammation results in the suppression of Sult1a1 and Sult2a1 gene expression [47,48].

An important cytokine that was downregulated in the OBO group is IL-17c, which plays a pivotal role in orchestrating both cellular and organismal metabolism in both physiological and pathogenic responses. Cellular metabolism profoundly influences the activity of IL-17 by modulating the differentiation and proliferation of Th17 cells. For instance, FAS in Th17 cells generates ligands favoring IL-17 production, while the balance between Th17 and Treg cells is determined by Fatty Acid Oxidation (FAO). Increased concentrations of IL-17 have been observed in various metabolic disorder scenarios, including obesity and diabetes [49,50]. Of interest is the IL-17c production by epithelia, which promotes inflammatory responses in the skin and the gut that is downregulated in the OBO cohort but requires further investigation since the Kyoto Encyclopedia of Genes and Genomes (KEGG) pathway analysis indicates a pro-inflammatory environment facilitating Inflammatory Bowel Disease (IBD), Systemic lupus erythematosus(SLE) and Rheumatoid Arthritis (RA)(Supplementary Figure S1D–F). Other proteins that were identified as downregulated in the OBO group remain underexplored in the context of their roles in metabolic syndrome and obesity, such as AXIN1, STAMBP, EIF4EBP1, CASP8, CA1, SOD1, GP1BA, NCAM1, KITLG, and ADA. For example, the mRNA and protein levels of AXIN1, a component of the β-catenin destruction complex, significantly increase during induced adipogenesis. This suggests a potential regulatory role for AXIN1 in adipocyte differentiation, warranting further exploration to elucidate its specific contributions to metabolic syndrome-related processes and obesity. Moreover, several proteins identified as downregulated in the OBO group, such as STAMBP, EIF4EBP1, CASP8, CA1, SOD1, GP1BA, NCAM1, KITLG, and ADA, represent novel findings that warrant further investigation. Notably, AXIN1, a key component of the β-catenin destruction complex, may play a crucial role in regulating adipocyte differentiation, suggesting potential contributions to metabolic syndrome and obesity that remain largely unexplored. These findings are significant, as few studies have focused on the proteomic differences between lean individuals and those with healthy obesity. For example, a recent study by Lieu et al. reported no significant proteomic differences between these groups [51], highlighting the novelty of our approach.

The characterized plasma protein profile in the OBO group compared to LHC in this study provides valuable insights into the molecular mechanisms associated with this unique obesity phenotype. The upregulation of key proteins such as IL-6, CXCL10, and CCL19 suggests an inflammatory signature in OBO individuals that may promote atherosclerosis along with other upregulated pro-inflammatory cytokines activating monocytes (Supplementary Figure S1C). Interestingly, the downregulation of NCAM1 and GP1BA may be protective in the context of microvascular clot formation, prompting further research in this direction to evaluate the CVS involvement in OBO individuals.

Functional enrichment analysis highlights the involvement of immunological pathways in obesity, underscoring the complex interplay between obesity and the immune system. The upregulation of pathways related to T cell differentiation, leukocyte differentiation, and inflammatory response suggests a potential link between obesity and immune dysregulation in OBO individuals. Furthermore, the enrichment of molecular components associated with chemoattractant and cytokine activity indicates a pro-inflammatory milieu in the plasma of OBO individuals.

Understanding the specific proteins and pathways affected by excess body fat in OBO individuals opens avenues for the development of targeted therapeutic interventions. By identifying these biomarkers before symptoms become evident, we may pave the way for preventive strategies to mitigate the progression of metabolic dysfunctions associated with obesity. We believe that further studies should be done wherein proteomic biomarkers need to be analyzed with respect to body fat distribution in individuals with obesity.

However, it is essential to acknowledge the limitations of our study. The major limitation of our study is the small size of the cohort analyzed, including its cross-sectional nature, and the need for longitudinal assessments to track changes over time. It is important to consider several individual factors that could influence the outcomes of this study, including lifestyle habits such as diet and exercise, as well as genetic predispositions, age, gender, and social status. Moreover, the overrepresentation of females in the obese group, relative to the non-obese group, may influence the results, as sex differences can affect metabolic outcomes. Additionally, metabolic health criteria, such as blood glucose and lipid levels, can vary across countries due to differences in clinical guidelines and population norms, potentially affecting the diagnostic consistency and clinical applicability of “obesity only” (OBO). All the observations were observed in serum rather than tissue; consequently, all these limitations should be considered when interpreting the findings. Therefore, further research is warranted to validate our findings in a larger multiethnic cohort and explore the long-term implications of the observed protein expression patterns in OBO individuals.

**Figure 4:**
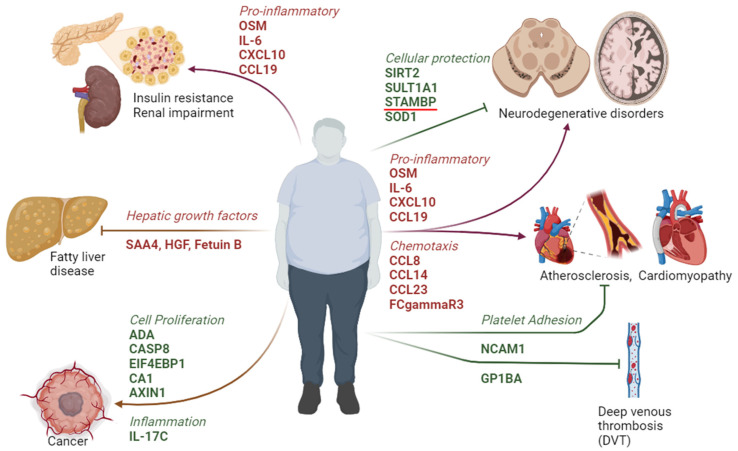
A schematic drawing representing the potential outcome of the metabolic signature and associated signaling pathways altered in OBO individuals.

The identification of proteomic signaling molecules aims to elucidate factors that may exhibit either protective or detrimental roles in individuals with clinically healthy obesity (OBO) as compared to lean healthy counterparts. The increase in inflammatory cytokines renders candidates susceptible to cardiovascular involvement, development of insulin resistance, and neurodegenerative disorders. Additionally, the downregulation of proteins involved in cellular protection and cell proliferation may predispose OBO individuals to cancer and diminished repair at the cellular level affecting multiple organ systems. However, other downregulated proteins that promote inflammation, cellular dysfunction, and coagulation confer protection in the OBO cohort (Figure 4). These findings suggest biomarkers and potential targets for intervention that necessitate additional mechanistic studies.

## 4. Materials and Methods

### 4.1. Study Population

The study was approved by the institutional review board (IRB) of Hamad Medical Corporation under study protocol (IRB protocol) #16245/16. The participants with normal body mass index (lean healthy control, LHC) or with obesity only (OBO) were recruited at the Qatar Metabolic Institute and provided written informed consent. For this study, we included men and women with BMI ≤ 25 kg/m^2^ for the LHC group and BMI ≥ 35 kg/m^2^ for the OBO group, who are generally healthy and free of any chronic illness. Fasting blood samples were collected, centrifuged at 1200× *g* at 4 °C for 10 min to isolate the serum, which was aliquoted, and stored at −80 °C until measured.

### 4.2. Measurements and Assays

Morning-fasted (at least 12 h) blood samples were collected in ethylenediaminetetraacetic acid (EDTA) tubes (Vacutainers; Becton Dickinson, Franklin Lakes, NJ, USA) and were centrifuged (1500× *g*) for 10 min to separate plasma. The plasma samples were stored at −80 °C until analysis. Blood biochemistry was performed at the HMC clinical laboratory. Measurements included HbA1c with turbidimetric inhibition immunoassay (TINIA Roche Diagnostics, Mannheim, Germany), glucose by the enzymatic reference method with hexokinase (Cobas 6000, Roche Diagnostics International, Basel, Switzerland). Total cholesterol, triglycerides, and HDL cholesterol levels were measured enzymatically using a Synchron LX20 analyzer (Beckman-Coulter, High Wycombe, UK).

### 4.3. Baseline Statistical Analysis

The baseline statistical analysis was performed to compute the mean, standard deviation, and *p*-value of the study participants for the OBO and LHC groups. Based on the type and distribution of the values for the computation of *p* values, different tests were used. A Shapiro–Wilk normality test was used to test the normality of the data. For normally distributed data, a Student’s *t*-test was performed, whereas a Mann–Whitney test was performed for the data not distributed normally.

For differential expression and pathway analysis, the R package version 3.28.0. 2023. was used. Differential expression analysis (Linear Models for Microarray Data [Limma]) was performed to identify differentially expressed proteins (DEPs) with more than a 1.25-fold change (0.32 log2 fold change) at FDR adjusted *p*-value of 0.05.

### 4.4. Proteomic Assay

Plasma inflammatory and cardiometabolic markers were assessed using Olink proximity extension assays (PEA) and 92-plex immunoassay (Uppsala, Sweden) following the standard protocol. In brief, the target protein binds to the double oligonucleotide-labeled antibody probe with high specificity, and then the microfluidic real-time PCR amplification of the oligonucleotide sequence is used to quantitatively detect the resulting DNA fragment [52]. Quality control and data normalization were carried out using the Normalized Protein eXpression (NPX) Manager software 2.2.1.311, and every run was checked and validated by the Olink support team in Uppsala, Sweden. Normalized Protein eXpression (NPX) values were provided as the final assay read-out. Olink data that did not pass quality control were excluded from analyses.

### 4.5. Bioinformatics

R packages in the standalone version of Integrated Differential Expression and Pathway analysis (iDEP) iDEP 0.96 (https://bioinformatics.sdstate.edu/idep/, accessed on 22 December 2023) [53], were used for data analysis. Briefly, customized hierarchical clustering (heatmap 2) was run with centered (subtracted mean) and normalized (divided by SD) samples and proteins. Differential expression analysis (Linear Models for Microarray Data [Limma]) was performed to identify differentially expressed proteins (DEPs) with more than a 1.25-fold change (0.32 log2 fold change) at FDR adjusted *p*-value of 0.05. A volcano plot summarized DEPs based on log2 fold changes across the two groups. Differential expression analysis accounted for obesity as the main effect, while correcting for age, sex, and race. Gene Ontology biological process (GO) and KEGG pathway enrichment analyses were conducted through the iDEP 0.96 data analysis platform.

Protein–protein interaction (PPI) network analysis was conducted using the STRING database v11.5 (https://string-db.org), as described before [54]. To obtain a comprehensive molecular characterization, data were integrated from the two Olink panels for analysis (Supplementary Data S1).

## 5. Conclusions

In conclusion, our results confirm that even though those subjects with obesity only (having no metabolic disease as such and considered metabolically healthy) have many aberrations in their proteome. These findings provide valuable insights into the underlying mechanisms, potentially paving the way for the identification of therapeutic targets aimed at improving metabolic health in individuals with obesity.

## Figures and Tables

**Table 1:** Clinical and biochemical traits of the study subjects: OBO (obesity only) and LHC (lean healthy controls). A Shapiro–Wilk normality test was used to test the normality of the data. For normally distributed data, a *t*-test was performed, whereas a Mann–Whitney test was performed for the data not distributed normally.

Feature	OBO (n = 27)	LHC (n = 15)	*p* Value
Age (years)	36.4 ± 5.5	38.8 ± 4.3	0.23
Gender (number)	17 (F) 10 (M)	7 (F) 8 (M)	0.004
Height (cm)	167.7 ± 11.3	172.2 ± 8.9	0.203
Weight (kg)	115.8 ± 21.5	73.6 ± 8.6	0.0004
BMI (kg/m^2^)	41.2 ± 4.9	24.8 ± 2.2	0.0002
Systolic Blood Pressure (mmHg)	118.0 ± 12.7	112.8 ± 10.7	0.197
Diastolic Blood Pressure (mmHg)	67.95 ± 13.2	68.0 ± 5.8	0.990
HbA1c (mmol/L)	35.7 ± 2.41	33.9 ± 1.86	0.069
Triglycerides (mmol/L)	1.19 ± 0.45	0.96 ± 0.35	0.143
Total Cholesterol (mmol/L)	4.62 ± 1.05	4.77 ± 0.99	0.665
Low-Density Lipoprotein Cholesterol (LDL-C) (mmol/L)	2.69 ± 1.07	2.96 ± 0.93	0.415
High-Density Lipoprotein Cholesterol (HDL-C) (mmol/L)	1.40 ± 0.59	1.35 ± 0.19	0.647
Glucose (mmol/L)	5.2 ± 0.7	4.7 ± 0.5	0.006
Creatinine (mmol/L)	65.9 ± 12.2	74.5 ± 13.3	0.041
Insulin (mIU/L)	20.0 ± 14.2	2.9 ± 1.7	0.0003
C-reactive Protein (CRP) (mg/L)	5.8 ± 1.8	1.3 ± 1.1	0.002
Alanine Aminotransferase (ALT) (U/L)	26.8 ± 18.5	23.5 ± 15.1	0.554
Aspartate Aminotransferase (AST) (U/L)	20.5 ± 9.4	21.1 ± 7.4	0.845

## Data Availability

All the data generated or analyzed during this study are included in this article and its Supplementary Materials. Further inquiries can be directed to the corresponding author.

## Supplementary Materials

The following supporting information can be downloaded at: https://www.mdpi.com/article/10.3390/ijms26052262/s1.
