# Supplementary figures and images for: Metabolically Healthy Obesity Is Characterized by a Distinct Proteome Signature
Source: Int J Mol Sci. 2025 Mar 4;26(5):2262. doi: 10.3390/ijms26052262 (PMC11901089; doi:10.3390/ijms26052262)

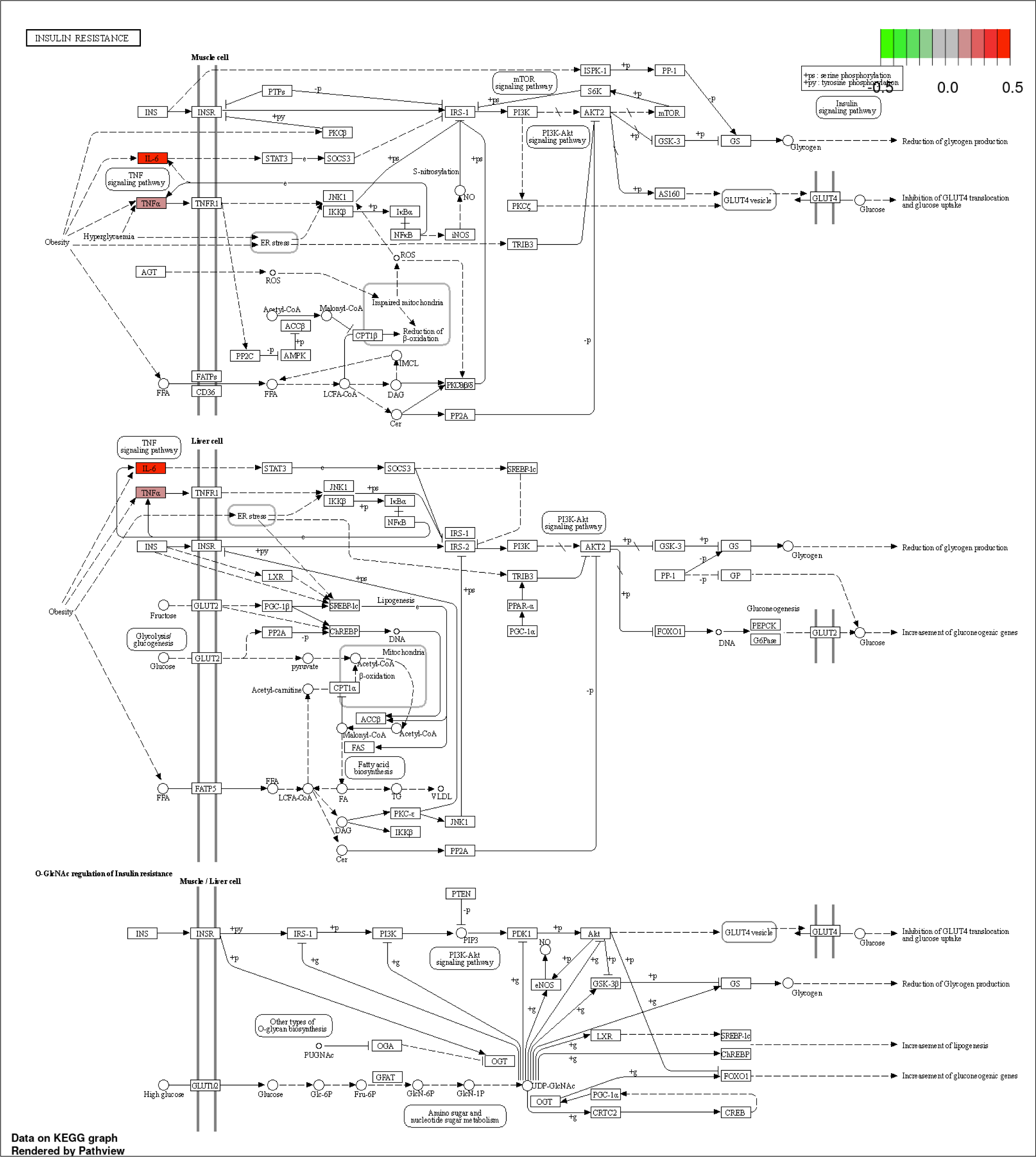

Supplement: Supplementary file 1 [file ijms-26-02262-s001.zip › Sup Figure S1-A.tif]

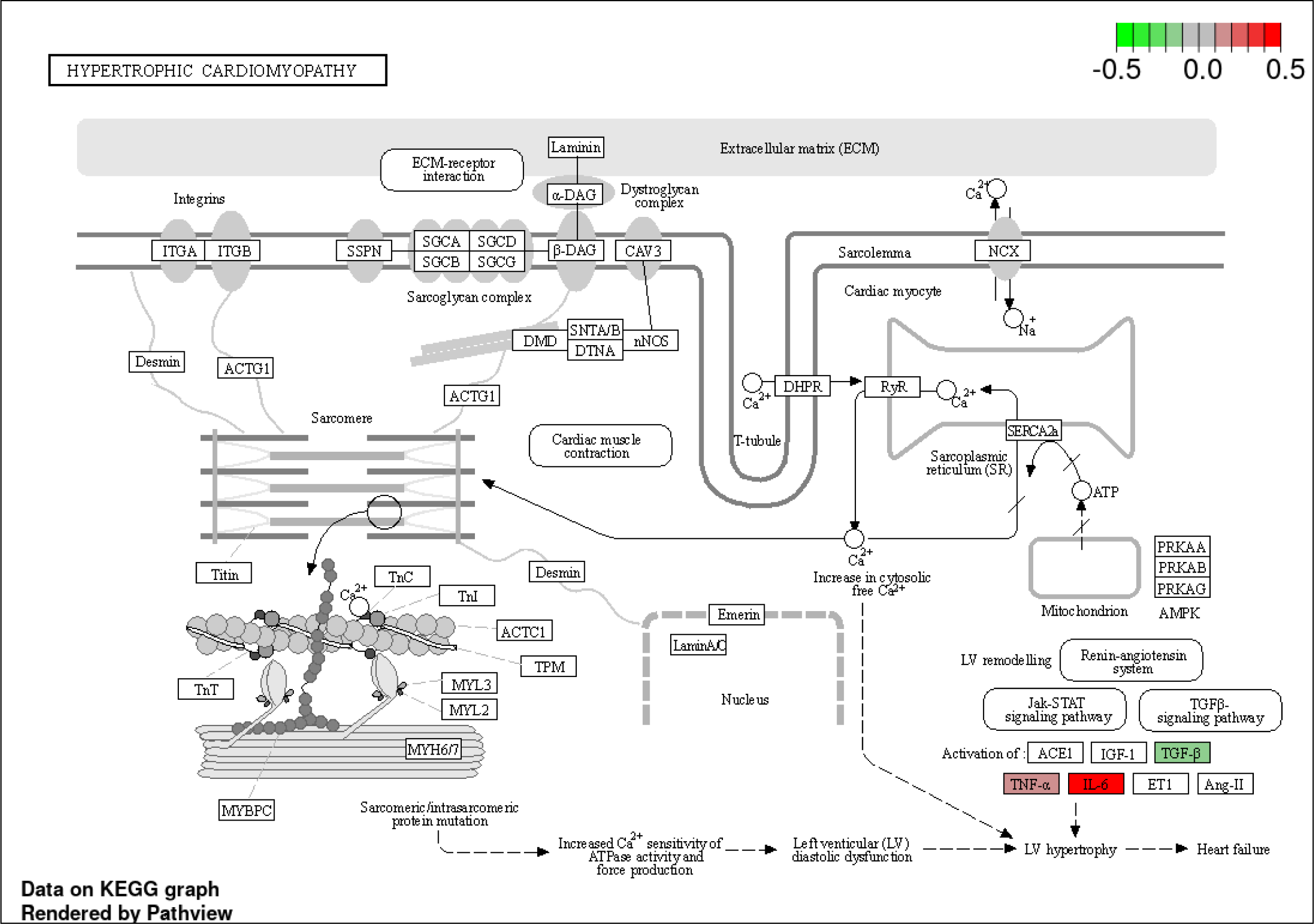

Supplement: Supplementary file 1 [file ijms-26-02262-s001.zip › Sup Figure S1-B.tif]

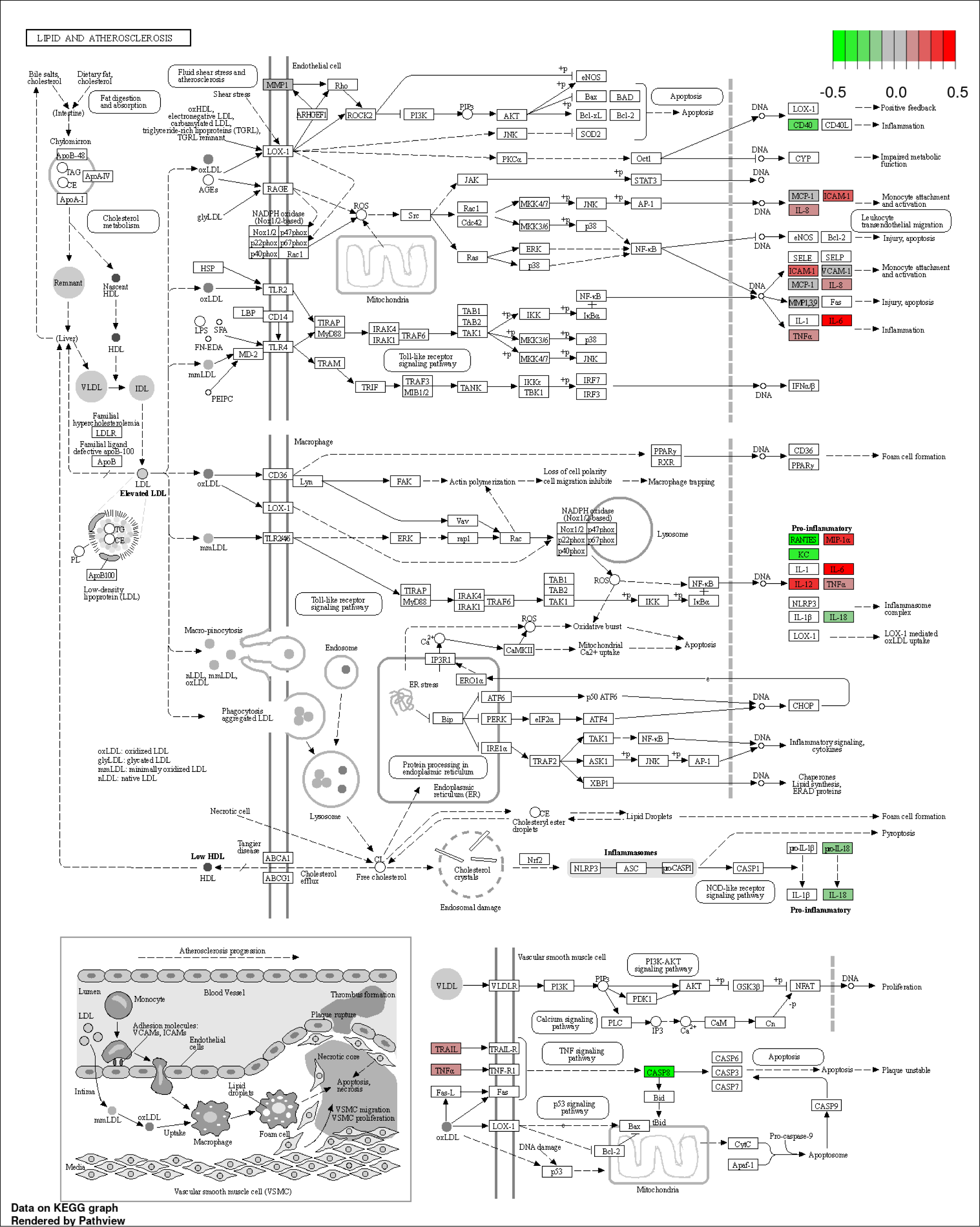

Supplement: Supplementary file 1 [file ijms-26-02262-s001.zip › Sup Figure S1-C.tif]

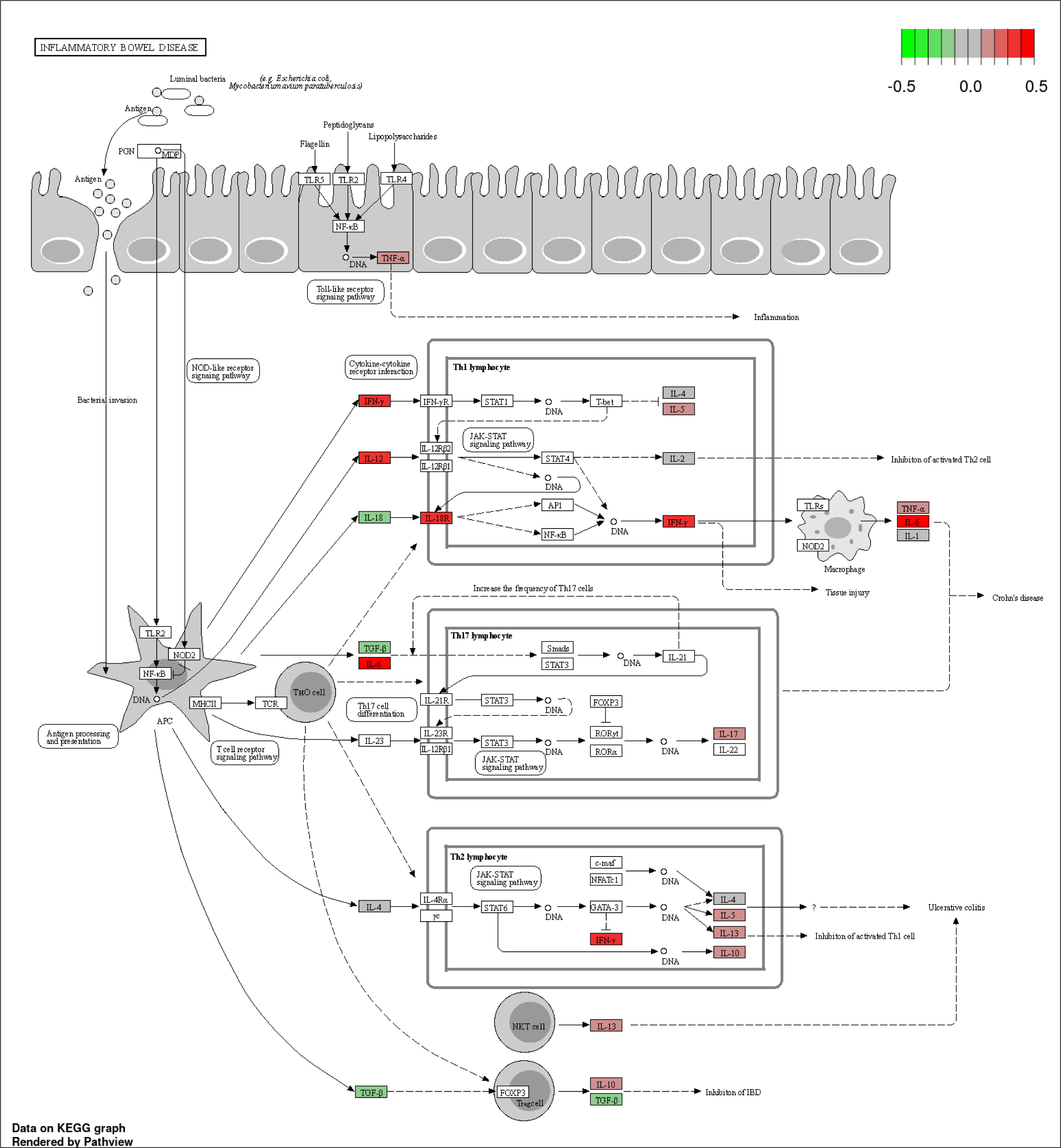

Supplement: Supplementary file 1 [file ijms-26-02262-s001.zip › Sup Figure S1-D.tif]

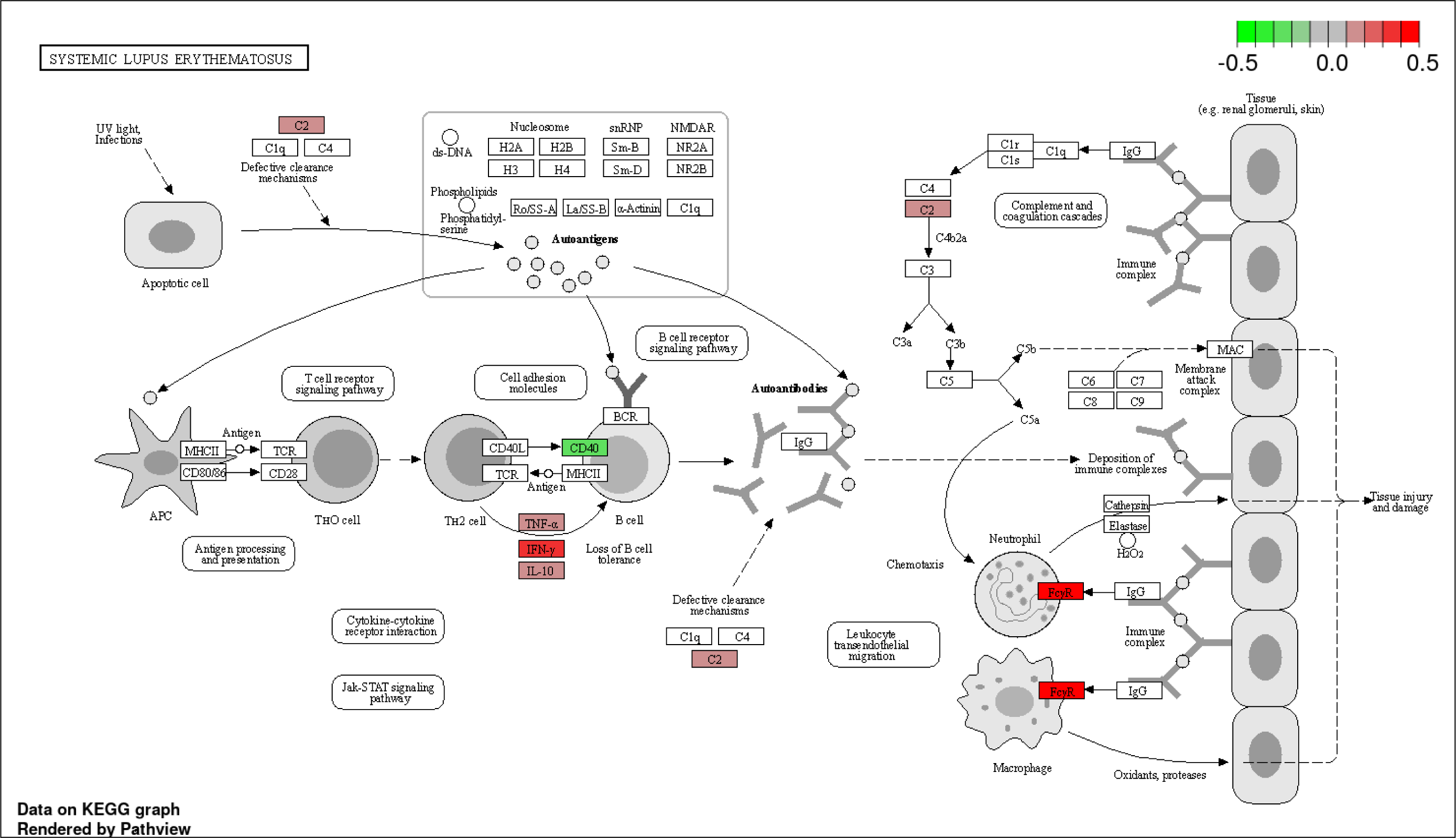

Supplement: Supplementary file 1 [file ijms-26-02262-s001.zip › Sup Figure S1-E.tif]

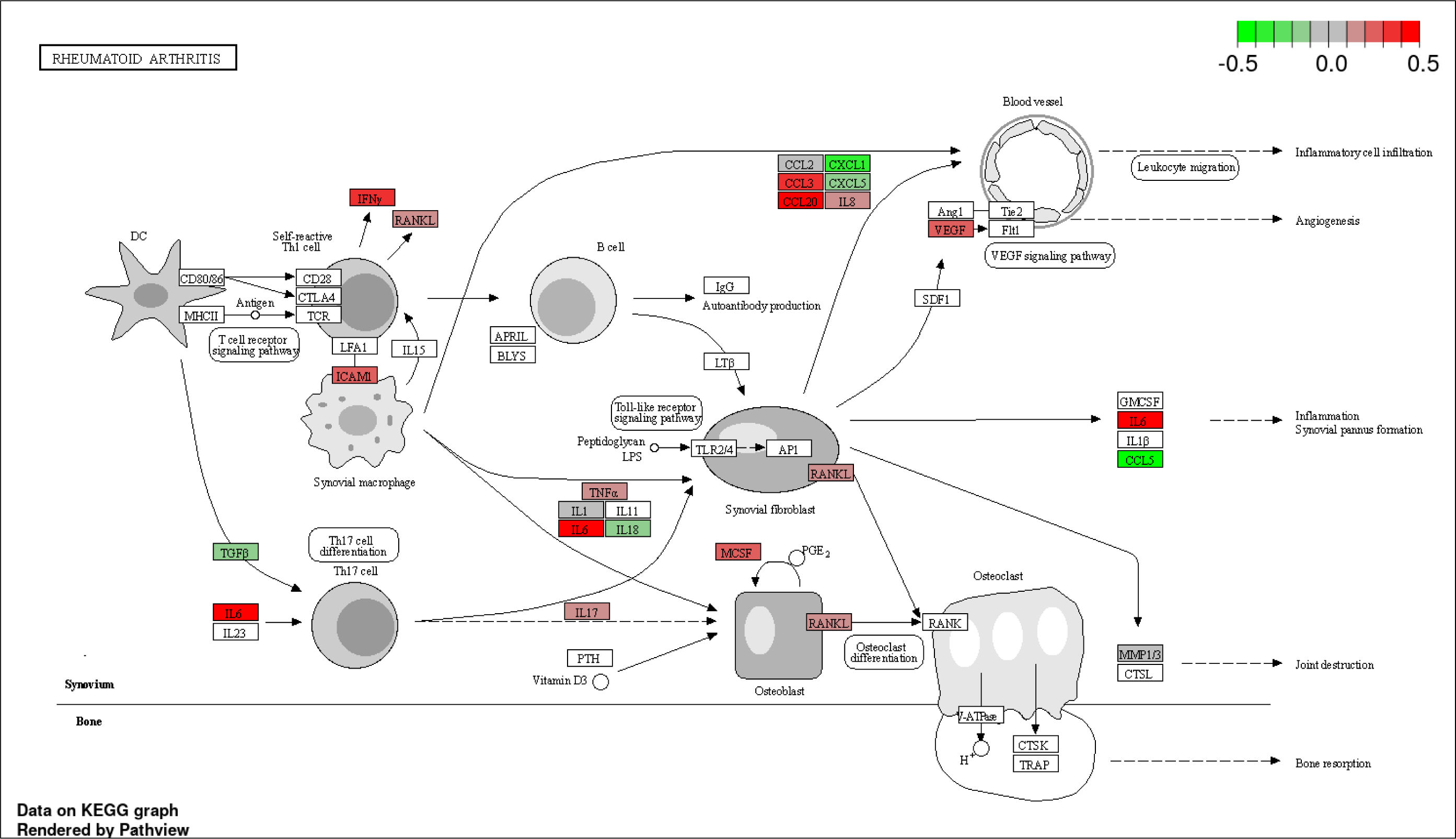

Supplement: Supplementary file 1 [file ijms-26-02262-s001.zip › Sup Figure S1-F.tif]

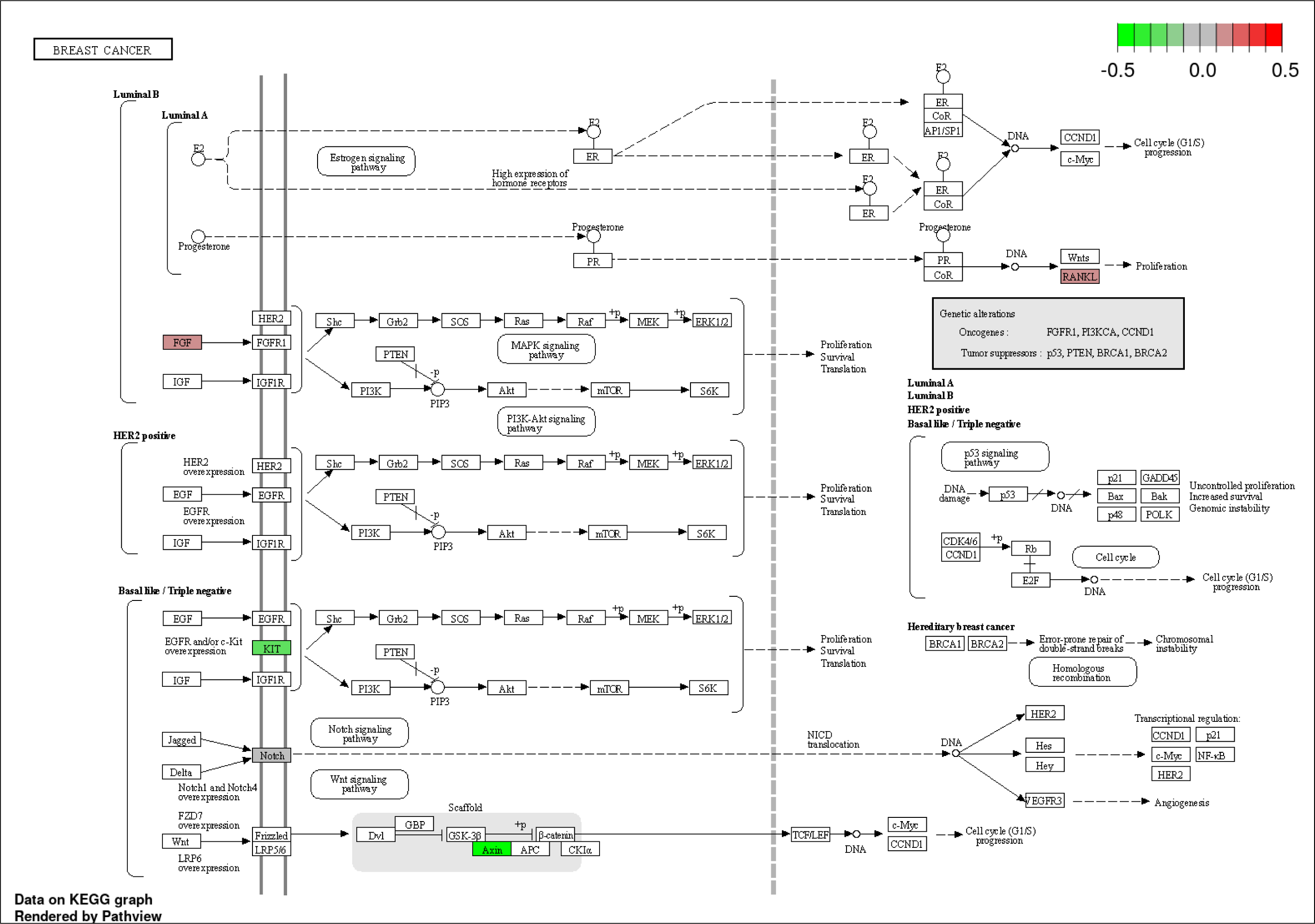

Supplement: Supplementary file 1 [file ijms-26-02262-s001.zip › Sup Figure S1-G.tif]

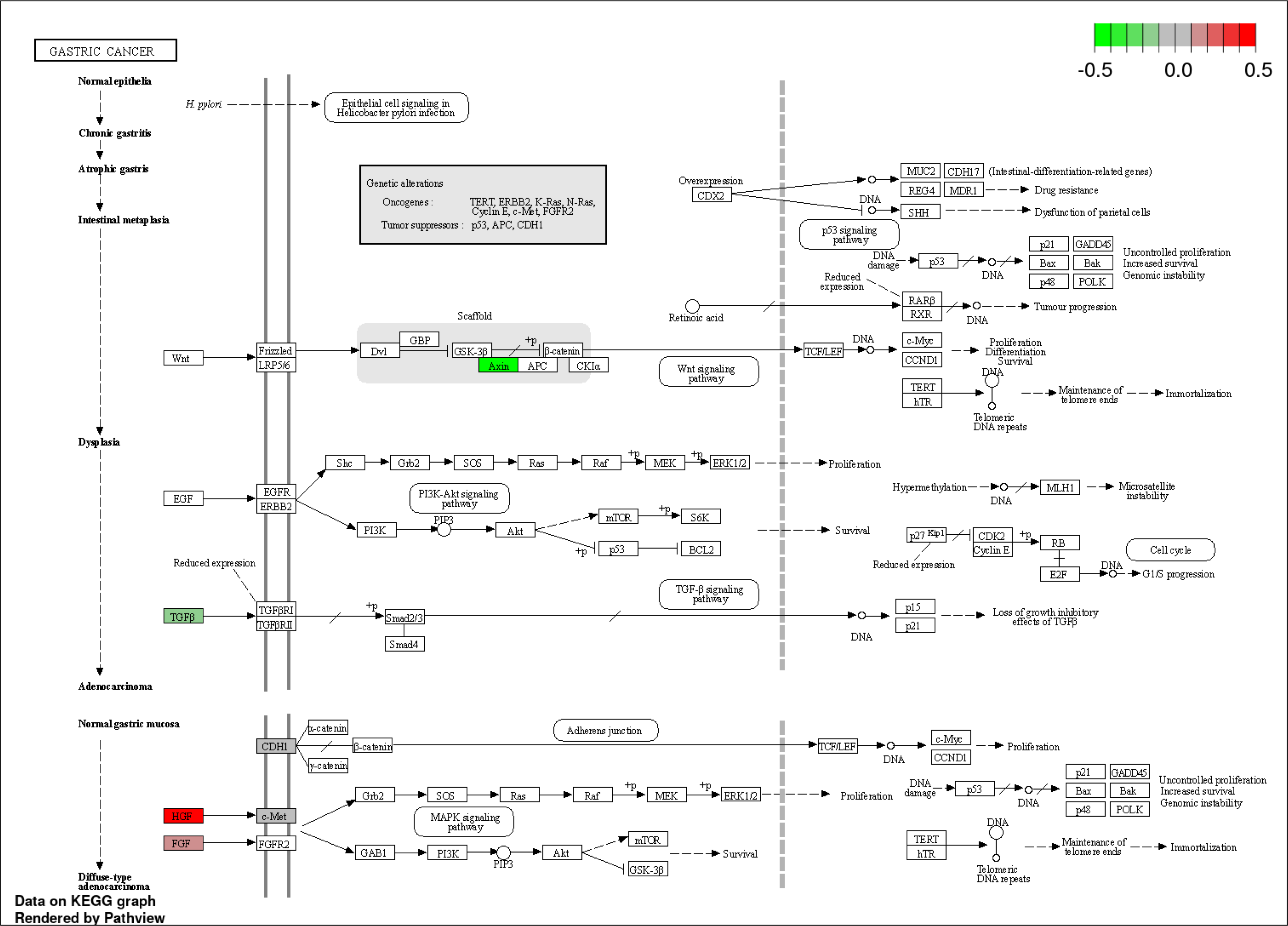

Supplement: Supplementary file 1 [file ijms-26-02262-s001.zip › Sup Figure S1-H.tif]

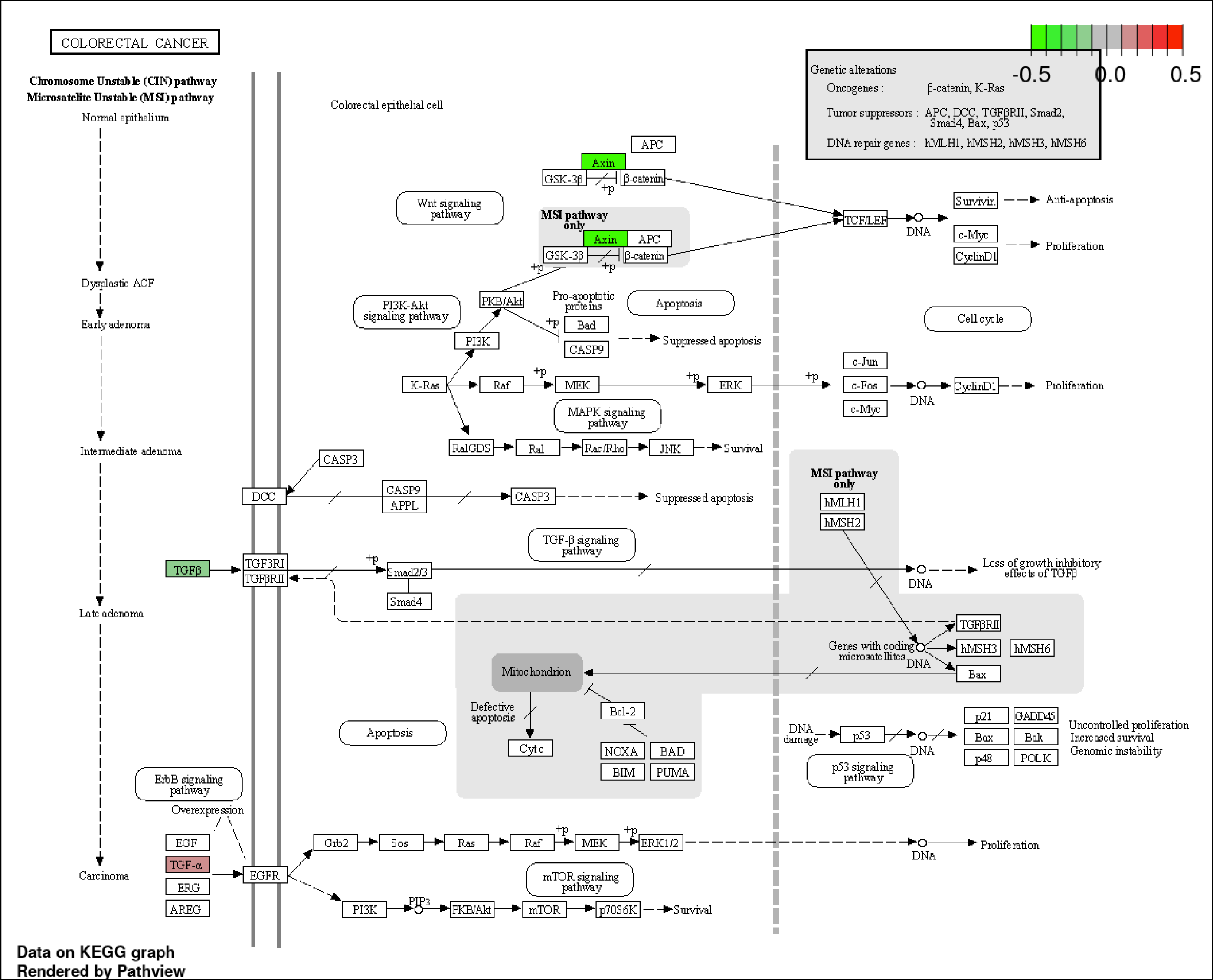

Supplement: Supplementary file 1 [file ijms-26-02262-s001.zip › Sup Figure S1-I.tif]

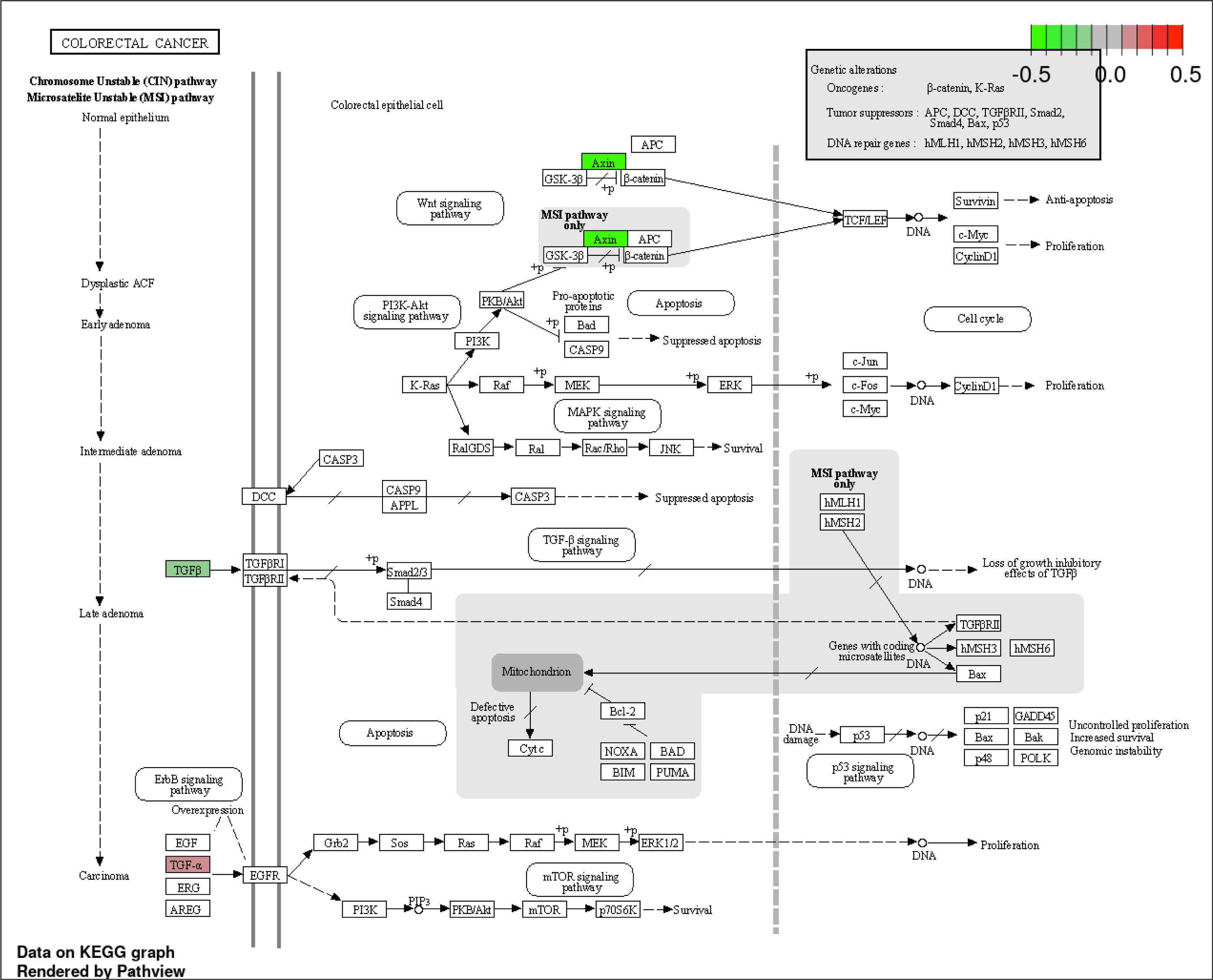

Supplement: Supplementary file 1 [file ijms-26-02262-s001.zip › Sup Figure S1-J.tif]

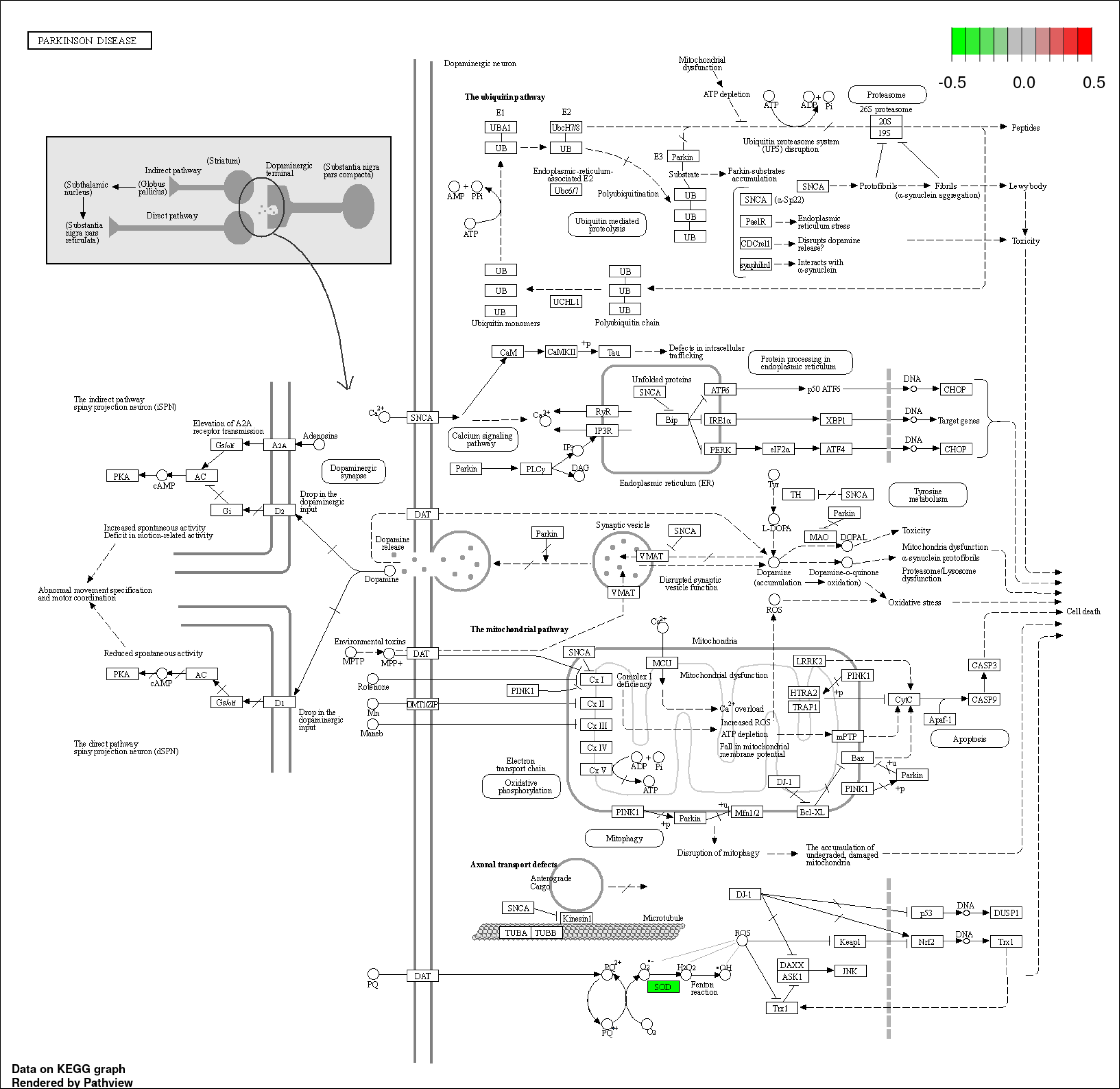

Supplement: Supplementary file 1 [file ijms-26-02262-s001.zip › Sup Figure S1-K.tif]

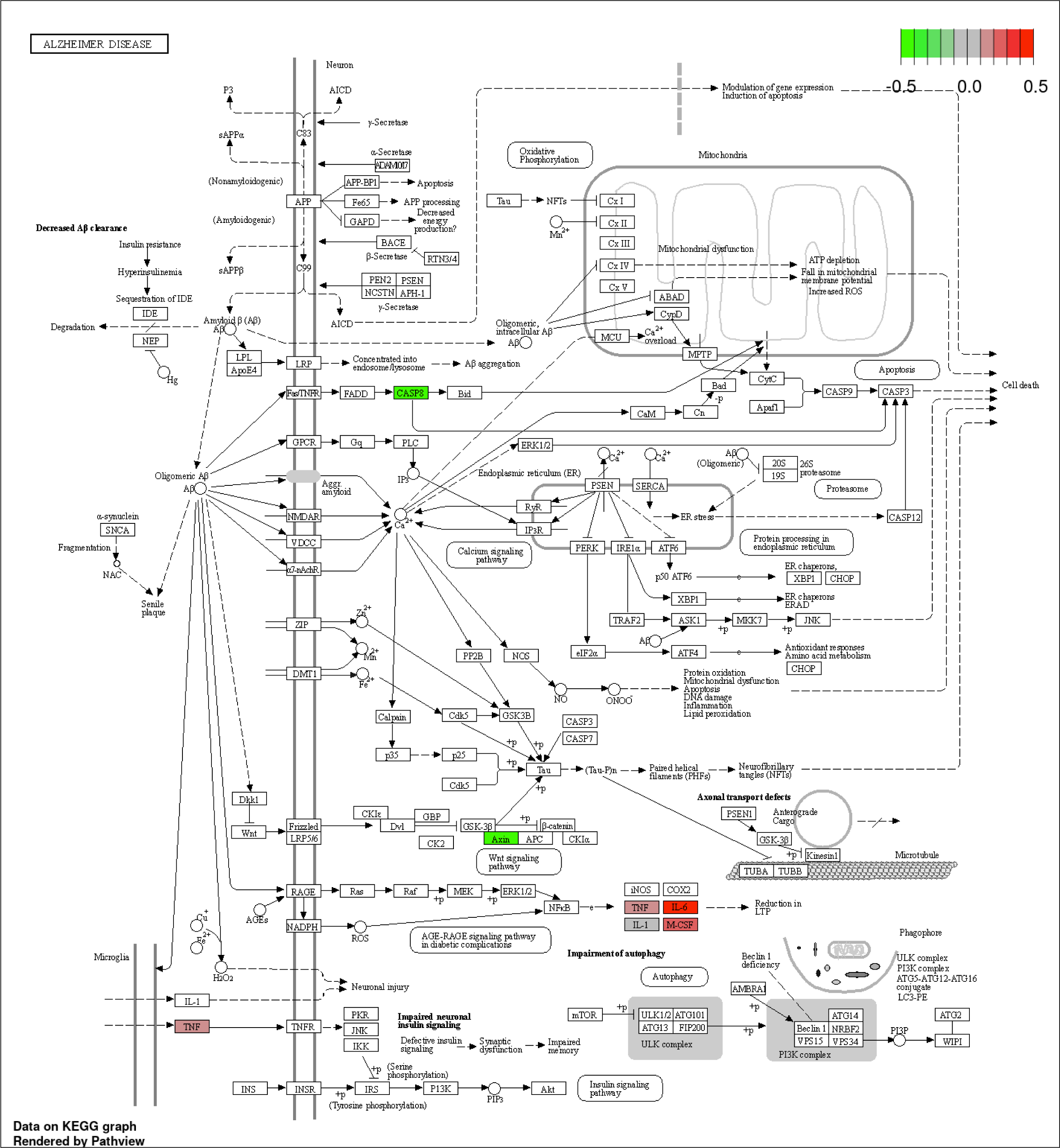

Supplement: Supplementary file 1 [file ijms-26-02262-s001.zip › Sup Figure S1-L.tif]

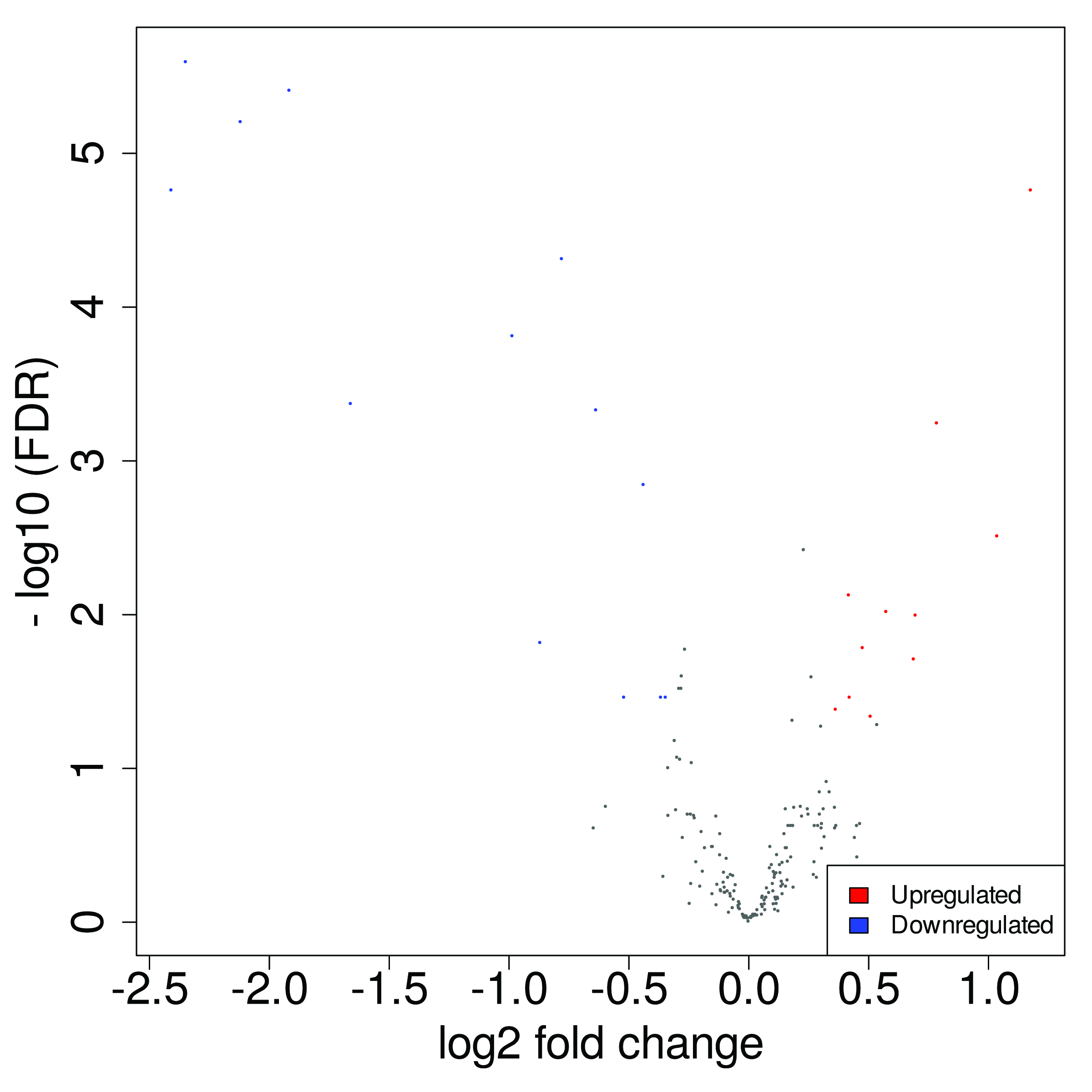

Supplement: Supplementary file 1 [file ijms-26-02262-s001.zip › Sup Figure S2 .tif]
